# Secondary Revision Surgery Using a Tensor Fascia Lata Sling Following Head and Neck Reconstruction

**DOI:** 10.1155/crid/2031645

**Published:** 2025-11-28

**Authors:** Hitoshi Nemoto, Kotaro Imagawa, Daiki Morita, Yukio Seki, Masahiro Uchibori, Takayuki Aoki, Masashi Sasaki, Yoshihide Ota

**Affiliations:** ^1^Department of Plastic Surgery, Tokai University School of Medicine, Isehara, Kanagawa, Japan; ^2^Department of Plastic and Reconstructive Surgery, Cancer Institute Hospital of the Japanese Foundation for Cancer Research, Tokyo, Japan; ^3^Department of Oral and Maxillofacial Surgery, Tokai University School of Medicine, Isehara, Kanagawa, Japan

## Abstract

**Background:**

Osteocutaneous flaps are the standard approach for performing reconstruction after a mandibulectomy. Soft tissue reconstruction alone may be performed for tumor progression or patient burden; however, it may cause a loss of oral function and issues concerning cosmetic appearance. We performed secondary revision surgery using the tensor fascia lata. This report presents a contour revision surgery for one case in which optimal flap selection was not possible during immediate reconstruction due to various reasons.

**Methods:**

A 63-year-old woman with idiopathic thrombocytopenic purpura and left lower gingival cancer (pT4a N3, M0, Stage IVa) underwent left hemimandibulectomy and left neck dissection. Reconstruction was performed with a deep inferior epigastric perforator flap. Two years after the surgery, we performed a flap volume reduction and created a tensor fascia lata sling to correct a significant irregularity of the mandibular contour. The mandibular contour was greatly improved after revision surgery, and controlled occlusion was possible. A quality-of-life assessment indicated an improved cosmetic appearance.

**Conclusion:**

This method is relatively simple and less invasive than bone grafting. It potentially solves some problems associated with mandibular reconstruction using only soft tissue.

## 1. Introduction

Several reconstruction methods are available for head and neck tumor ablation with mandibulectomy, including using osteocutaneous flaps (e.g., fibula), titanium mandibular reconstruction plates and soft tissue flaps, and soft tissue flaps alone [1, 2]. In such reconstructions, using an osteocutaneous flap is preferred considering the patient's postoperative function and cosmetic appearance [2–4]; however, soft tissue flap reconstruction alone may be used depending on the defect size, the degree of tumor progression, and the patient's condition [1]. Reconstruction using only soft tissue will typically cause a decline in oral function and cosmetic appearance, affecting the patient's quality of life [4]. Revision surgery is considered for cases without metastasis or recurrence. Although osteocutaneous flap grafting is considered a revision procedure, it is relatively invasive, and some patients choose to decline the procedure. In addition, scar contracture caused by surgery or radiation may make restoring aesthetic appearance and oral function challenging, even with revision surgery using osteocutaneous flap grafting.

Very few reports have been published on secondary revision surgery in these cases. We report a case in which a relatively simple and less invasive secondary revision procedure was performed using a tensor fascia lata (TFL) sling. This report presents a contour revision surgery for one case in which optimal flap selection was not possible during immediate reconstruction due to various reasons. This article was written in accordance with CARE guidelines.

## 2. Case Presentation

**Figure 1:**
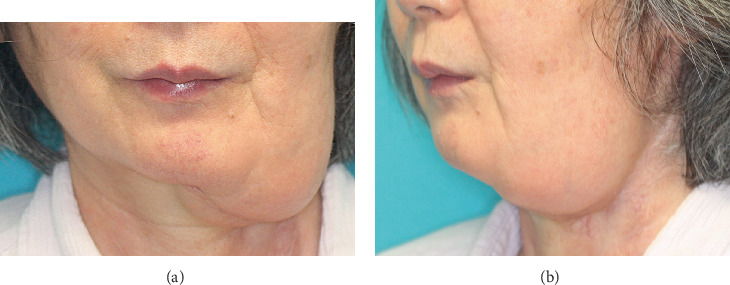
(a, b) Two years after reconstruction. A 63-year-old woman with idiopathic thrombocytopenic purpura underwent left hemimandibulectomy and resection of the masticatory muscles for left gingival cancer and soft tissue–only reconstruction with a free deep inferior epigastric perforator flap. Two years after surgery, an irregularity of the mandibular contour is observed, resulting from significant flap drooping.

A 63-year-old woman with idiopathic thrombocytopenic purpura and a body mass index of 24.3 kg/m^2^ was diagnosed with left lower gingival cancer (cT3, N2b, M0, Stage IVa). She underwent left hemimandibulectomy including infratemporal fossa dissection and left neck dissection. Reconstruction was performed with a deep inferior epigastric perforator flap that was 7 × 15 cm in size. End-to-end anastomosis was performed to connect the flap artery to the superior thyroid artery and the flap vein to the internal jugular vein. The operation lasted 8 h and 2 min, with 856 mL of blood loss. The flap survived uneventfully. The surgical margins were negative. Although metastasis was present in four cervical lymph nodes, no extranodal invasion was observed. Thus, postoperative chemoradiotherapy was not performed. The postoperative stage was pT4a, N3, M0, Stage IVa. After surgery, the transplanted flap drooped, and a significant irregularity of the mandibular contour was observed (Figure 1), and because of this drooping, the patient avoided going out as much as possible. The patient also complained of malocclusion and having difficulty chewing. Two years passed without recurrence, and revision surgery was performed. Preoperative blood tests showed that the platelet counts were maintained at 76,000 per milliliter and, based on the expected blood loss, we determined that the patient could tolerate secondary revision surgery.

**Figure 2:**
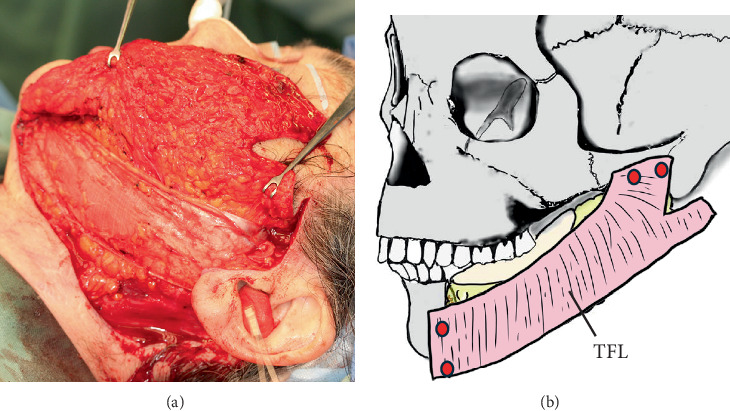
(a) Tensor fascia lata sling. (b) Surgical schema. After reducing the flap volume, a 7 × 15cm section of the tensor fascia lata, harvested from the left thigh, supports the drooping flap like a hammock. The zygomatic arch and residual mandible are fixed with a Mitek QuickAnchor Plus device (DePuy Mitek Inc., Raynham, Massachusetts, United States; red circles). The superior part of the tensor fascia lata is separated to avoid the auricle and sutured to the periosteum of the mastoid process.

**Figure 3:**
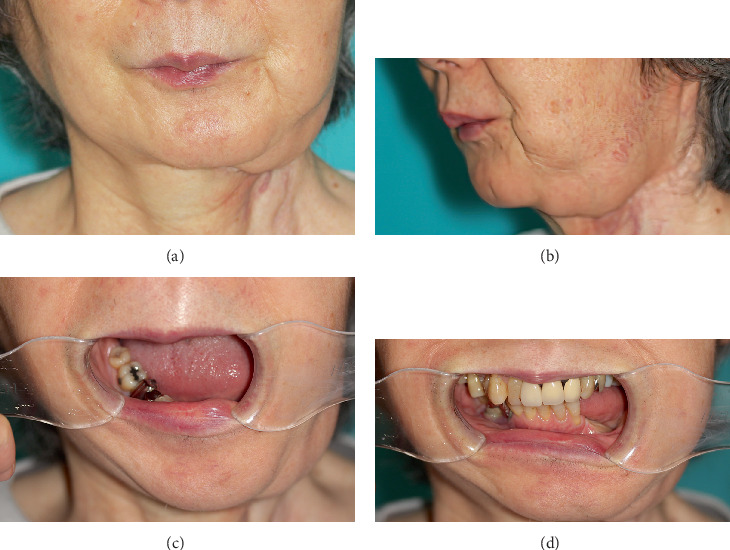
One year after secondary revision surgery. (a, b) The contour of the mandible is improved. (c, d) When opening the mouth, the residual mandible is deviated to the affected side; however, controlled occlusion is possible.

**Figure 4:**
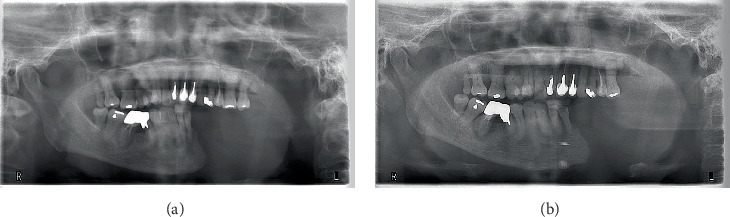
Orthopantomogram of the mandible. (a) Before secondary revision surgery. Hemimandibulectomy was performed between the left lower first and second teeth. (b) After secondary revision surgery. Two Mitek QuickAnchor Plus devices are visible at the margin of the mandibular resection. The mandibular position remains largely unchanged from the preoperative position, and no deterioration in occlusion is observed.

**Figure 5:**
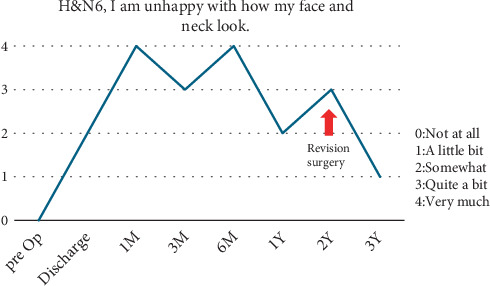
Quality of life assessment, conducted using the Functional Assessment of Cancer Therapy–Head and Neck Scale (Version 4). After revision surgery, improvements are observed in the item involving cosmetic appearance (H&N6).

The revision surgery was performed under general anesthesia with the patient in the supine position. The scar from the previous left neck dissection was incised, and the flap's fat was removed after subcutaneous dissection, taking care not to injure the transplanted flap's pedicle. After removing the scar tissue and increasing the mobility of the remaining mandible, a 7 × 15 cm piece of the left TFL was harvested and attached to the zygomatic arch and the remaining mandible using a Mitek QuickAnchor Plus (DePuy Mitek Inc., Raynham, Massachusetts, United States). This TFL piece supported the drooping transplant flap like a hammock (Figure 2). The superior part of the TFL was separated to avoid the auricle and sutured to the periosteum of the mastoid process. Intermaxillary fixation with a hybrid MMF (Stryker Corporation, Portage, Michigan, United States) was performed simultaneously because this procedure would cause traction of the residual mandible on the affected side. A Z-plasty was also performed to treat scar contracture in the oral cavity. The surgery lasted 4 h and 18 min. After surgery, the patient continued with intermaxillary fixation between meals and wore a facial garment for 3 months. The patient was highly motivated to achieve improvement and was able to complete these postoperative care measures. The postoperative course was uneventful; the mandibular contour improved significantly after the revision surgery, and controlled occlusion was possible (Figures 3, 3, 3, 3, and 4). The patient was happy with the outcome because she could now go out without worrying about her appearance. Improvements were observed in the postoperative cosmetic appearance item of the Functional Assessment of Cancer Therapy–Head and Neck Scale Score (Version 4) [5] 1 year after the revision surgery (Figure 5).

## 3. Discussion

The fibula flap is often used to reconstruct a large area of the mandible [2]. The advantage of using the fibula flap is that it can be harvested from a long bone and is easy to process. However, compared to a free soft tissue flap, the harvesting and processing take longer, donor site morbidity is relatively common, and the flap has less soft tissue [1]. In cases with advanced progression of tumor, the resection often extends into the infratemporal fossa (infratemporal fossa dissection), with extensive tissue defects occurring in the infratemporal fossa following total resection of the mandibular condyle including the temporomandibular joint capsule and disc, the masticatory muscle groups (lateral pterygoid and medial pterygoid muscles), and the muscles' origin (the lateral pterygoid plate, the pterygoid process, and the greater wing of the sphenoid bone). Furthermore, the presence of the pterygoid venous plexus [6] requires that the soft tissue be tightly packed to fill the dead space and prevent postoperative bleeding and infection. Thus, reconstruction after infratemporal fossa dissection requires a greater volume of tissue. Moreover, the effects of postoperative radiotherapy on the transplanted bone and the complications associated with the metal plates used for bone fixation need to be considered [7, 8]. Ideally, cases with large defects require a double free flap, in which a free flap (e.g., an anterolateral thigh flap) is added to the fibula flap. However, only a limited number of institutions can perform this procedure because of increased operative time and requirements for surgical staffing [9]. The indication for soft tissue–only reconstruction after a mandibulectomy depends on the patient's level of burden or the extent of the soft tissue defect. In the present case, the patient had thrombocytopenia and was at high risk for bleeding; almost half of the mandible and the masseter muscle were to be resected, and in addition, infratemporal fossa dissection was performed. Therefore, we determined that a fibula flap alone would be insufficient to fill the dead space, and reconstruction was performed using only a free soft tissue flap. In the present case, the deep inferior epigastric perforator flap was selected because adequate tissue transfer to the infratemporal fossa was considered crucial.

In cases of soft tissue–only reconstruction, malocclusion may occur due to postoperative deviation of the remaining mandible on the affected side [1]. Deviations can be caused by the lack of a countermandible, postoperative scar contracture, or traction arising from the transplanted flap drooping, shrinking, and small size. In particular, flap droop significantly affects facial contours, and facial disfigurement is a major factor in reducing a patient's quality of life [10]. Flap droop is caused by insufficient flap support and excessive flap volume. Therefore, we adopted strategies to minimize the flap weight by defatting and grafting the TFL to support the flap. At this time, the available data on secondary revision surgery in these cases is not sufficient to determine whether flap debulking surgery alone can yield favorable outcomes. Therefore, we sought to achieve more certain results by adding the TFL grafting method. This TFL sling may cause further deviation of the residual mandible on the affected side; however, intermaxillary fixation and appropriate tension of the TFL may prevent further deterioration in occlusion. In addition, these strategies improved the contour of the mandible and facilitated pulling the remainder of the mandible toward the healthy side by supporting the flap, allowing controlled occlusion. The absolute prerequisite for controlled occlusion is that the flap is large enough. Thus, a larger flap should be used for soft tissue–only reconstruction, considering the shrinkage that occurs postoperatively when a free flap transfer is performed. For mild facial contour deformities, debulking with liposuction and lipoinjection may be sufficient [11, 12].

The rate of shrinkage of flap volume is approximately 30% after surgery [13, 14]. However, because postsurgical changes in the patient's weight are unpredictable, thinning the flap in advance is discouraged when performing a free flap transfer. Furthermore, the appropriate flap thickness for such cases remains unclear.

By incorporating a long TFL into the anterolateral thigh flap and supporting the flap during reconstruction, as we performed in our method, flap drooping may be prevented. Attaching a large amount of fascia to the rectus abdominis flap is expected to increase the incidence of abdominal wall hernia. However, it is important to consider the potential benefits of this approach, including the possibility of eliminating the need for subsequent secondary revision surgery. This could be a valuable option to consider during immediate reconstruction. The TFL can be elevated and transferred with its vascular pedicle. A vascularized TFL offers advantages in preventing contracture and infection. However, this procedure requires dissection of the vascular pedicle and vascular anastomosis, inevitably prolonging the surgical time. In cases where the use of a free flap is feasible, the fibular flap should be the preferred choice. The patient in the present case presented with concomitant idiopathic thrombocytopenic purpura, necessitating a minimally invasive approach to achieve symptom improvement. Harvesting and transplanting a nonvascularized TFL is straightforward but carries a risk of contracture and may become a source of infection. We believe that performing the surgical procedure transcutaneously, rather than intraorally, results in a lower infection rate. Furthermore, although contracture of the TFL is possible, we consider that contracture may increase support to the tissue of the cheek, which benefits the facial contour. Although contracture could potentially worsen displacement of the remaining mandible, that this can be prevented with postoperative intermaxillary fixation seems possible.

The authors believe that osteocutaneous flap reconstruction is preferable for head and neck tumor resection with mandibulectomy. However, when soft tissue reconstruction alone is unavoidable, secondary revision surgery using this method can be expected to improve facial contours without worsening oral function.

To conclude, in the present case, a patient presented with facial contour deformity and reduced oral function after reconstruction using soft tissue alone following resection of a head and neck tumor with mandibulectomy. Secondary revision surgery with a volume reduction of the flap and a TFL sling improved the facial contour without worsening oral function. This method is relatively simple and less invasive than bone grafting and potentially solves some of the problems associated with mandibular reconstruction using only soft tissue.

## Data Availability

The data that support the findings of this study are available from the corresponding author upon reasonable request.
